# Arabidopsis *OST1* homologs of barley are involved in stomatal regulation

**DOI:** 10.1093/jxb/erag037

**Published:** 2026-01-22

**Authors:** Kajal Samantara, Eliisabeth Laul, Egon Meigas, Isabella Siil, Mikk Välbe, Hannes Kollist, Kristiina Laanemets, Dmitry Yarmolinsky, Ebe Merilo

**Affiliations:** Institute of Technology, University of Tartu, Nooruse 1, Tartu 50411, Estonia; Centre of Estonian Rural Research and Knowledge, J. Aamisepa 1, Jõgeva 48309, Estonia; Institute of Technology, University of Tartu, Nooruse 1, Tartu 50411, Estonia; Institute of Technology, University of Tartu, Nooruse 1, Tartu 50411, Estonia; Institute of Technology, University of Tartu, Nooruse 1, Tartu 50411, Estonia; Institute of Bioengineering, University of Tartu, Nooruse 1, Tartu 50411, Estonia; Centre of Estonian Rural Research and Knowledge, J. Aamisepa 1, Jõgeva 48309, Estonia; Institute of Bioengineering, University of Tartu, Nooruse 1, Tartu 50411, Estonia; Institute of Technology, University of Tartu, Nooruse 1, Tartu 50411, Estonia; ETH Zürich, Switzerland

**Keywords:** Abscisic acid, barley, grain yield, stomatal density, stomatal regulation, vapor pressure deficit

## Abstract

Stomata on leaves determine water loss from plants and carbon uptake for photosynthesis. These key physiological processes affect biomass production, stress sensitivity and water use efficiency in plants, and are also addressed in breeding programs. Abscisic acid (ABA) is an important plant stress hormone, whose signaling module described for Arabidopsis is conserved in land plants: it controls the activity of protein kinase OST1 (Open Stomata 1, also SnRK2.6/SRK2E), which triggers stomatal closure by activating guard cell anion channels. Here, we generated barley double mutants defective in proteins homologous to Arabidopsis OST1 and measured their stomatal conductance and responses to ABA spraying and rising vapor pressure deficit (VPD). These barley mutants showed wider stomatal apertures, higher stomatal conductances and decreased VPD- and ABA-sensitivities compared with wild-type (‘Golden Promise’) in gas exchange experiments, whereas their net assimilation rates and stomatal densities were only mildly affected. No statistically significant difference between double mutants and wild type was detected in grain yield per plant under well-watered conditions. These results show that OST1-like kinases HvSnRK2.7 and HvSnRK2.9 control stomatal conductance and sensitivity to ABA and VPD in barley. The effect of modified ABA signaling pathway on stress tolerance and yield is discussed in the context of breeding plants that are productive under various conditions of water availability.

## Teaser

Barley double mutants defective in proteins homologous to Arabidopsis OST1 showed enhanced stomatal conductance and decreased stomatal responsiveness to low air humidity and abscisic acid.

## Introduction

The phytohormone abscisic acid (ABA) controls plant transpiration through stomata, water-use efficiency and drought tolerance in various conditions. Subgroup III SNF1-related protein kinase 2s (SnRK2s: SnRK2.2/SRK2D, SnRK2.3/SRK2I and SnRK2.6/SRK2E) are positive regulators of ABA signaling in Arabidopsis: SRK2D and SRK2I are expressed in leaves and roots, whereas SRK2E (also named OPEN STOMATA 1, OST1) is strongly expressed in guard cells (Fujita *et al*., 2009). In barley, a total of 10 *SnRK2* genes have been identified; the ABA responsive elements were predicted to be present in the promoters of eight of these (all except SnRK2.8 and SnRK2.10; Chen *et al.*, 2021). Under favorable conditions with low ABA levels, protein phosphatases type 2C (PP2Cs) repress the activity of SnRK2.2, SnRK2.3 and SnRK2.6, keeping the ABA signaling pathway inactive. ABA biosynthesis and binding to its PYRABACTIN RESISTANCE1 (PYR1), PYR1-LIKE (PYL), REGULATORY COMPONENTS OF ABA RECEPTORS (RCAR) (PYR/PYL/RCAR) receptors sequesters PP2Cs, resulting in activation of SnRK2s and downstream responses (Ma *et al*., 2009; Park *et al*., 2009). Arabidopsis loss-of-function mutants for SnRK2.6/SRK2E/OST1 are severely impaired in stomatal regulation: *ost1-3* plants display strongly decreased stomatal closure to exogenous ABA, high atmospheric vapor pressure deficit (VPD), and ozone, whereas their responses to darkness and elevated CO_2_ concentration are delayed (Mustilli *et al*., 2002; Xue *et al*., 2011; Merilo *et al*., 2013; Jalakas *et al*., 2018a). Under well-watered conditions, stomatal response of *ost1-3* plants to high VPD is severely impaired (Xie *et al*., 2006; Merilo *et al*., 2013, 2018; Hsu *et al*., 2021; Jalakas *et al*., 2021). However, when grown under soil water deficit, stomata of *ost1-3* plants were VPD-sensitive (Tulva *et al*., 2023). Stomatal development, on the other hand, is OST1-independent in Arabidopsis (Jalakas *et al*., 2018a).

In Arabidopsis, OST1 is involved in both stomatal and whole-plant water status regulation. Among 1656 unique proteins identified as putative phosphorylation substrates for OST1 were known regulators of ABA signaling, proteins involved in osmotic stress responses, but also in vesicle trafficking, regulation of gene expression, etc. (Wang *et al*., 2020). OST1 was found to phosphorylate guard cell Plasma membrane Intrinsic Protein 2;1 (PIP2;1) aquaporin, enhancing its water transport activity and promoting stomatal closure (Grondin *et al*., 2015). Furthermore, OST1 phosphorylated Arabidopsis Chloride Channel AtCLCa, a dual function anion/proton exchanger of guard cells, which resulted in increased outward-directing anion fluxes across the tonoplast, necessary for stomatal closure (Wege *et al*., 2014). Since OST1 also activates plasma membrane anion channels (Geiger *et al*., 2009; Imes *et al*., 2013), Wege *et al*. (2014) proposed that OST1 might coordinate the fluxes of ions across the tonoplast and plasma membrane of guard cells during stomatal closure. In relation to water uptake, root growth regulation during drought is also mediated by SnRK2s, which activate different sucrose transporters (SWEETs) for sucrose export from leaves to roots (Chen *et al*., 2022; Chong *et al*., 2022a).

Besides its important role in controlling plant hydraulics, OST1 has many other functions in plants. As an example, OST1 positively regulates cold tolerance in *Arabidopsis thaliana* and rice (Ding *et al*., 2015, 2018; Wang *et al*., 2021). In tomato (*Solanum lycopersicum*), SlOST1 promotes flowering under drought stress (Chong *et al*., 2022b). OST1 activity also influences plant growth, although this effect can be species-specific. The *slost1* tomato mutants were smaller than the wild type plants (Chong *et al*., 2022b). In Arabidopsis, *ost1-3* mutants showed no growth penalty in terms of rosette area or rosette dry weight, compared with wild type under either low or high VPD conditions at the final harvest; rather, *ost1-3* plants were larger throughout the experiment (Tulva *et al*., 2023). SnRK2 kinases were found to control growth of Arabidopsis via SNF1-related protein kinase 1 (SnRK1) and target of rapamycin (TOR) (Belda-Palazón *et al*., 2020). In barley (*Hordeum vulgare*) grains, the OST1 homolog ABA-induced protein kinase (PKABA) was reported to participate in ABA signaling (Gomez-Cadenas *et al*., 1999).

One of the most important climate change-associated plant stresses is rising atmospheric VPD, the difference between the saturation and actual air vapor pressures (Ficklin and Novick, 2017; Vicente-Serrano *et al*., 2018; Grossiord *et al*., 2020). Rising VPD results in decreased stomatal conductance, photosynthesis, leaf area and, finally, in lower growth and yield (Lopez *et al*., 2021). Generally, growth penalties under high VPD have been linked to decreased stomatal conductance and photosynthesis (Grossiord *et al*., 2020). Here, we generated barley lines with knocked out HvSnRK2.7/SnRK2.9 to translate Arabidopsis-based knowledge about the role of OST1 in stomatal regulation to crops. We hypothesized that barley *snrk2.7 snrk2.9* double mutants have larger stomatal conductance and impaired stomatal regulation, as well as higher net CO_2_ assimilation rate and grain yield than wildtype, in well-watered conditions.

## Materials and methods

### Identification of AtOST1 homologs in the barley genome and generation of Arabidopsis complementation lines

Using the protein sequence of Arabidopsis OST1, a BLAST search in the published barley genome available at Phytozome (https://phytozome-next.jgi.doe.gov/) was performed. As a result, two proteins with high similarity to Arabidopsis OST1 were identified, corresponding to HvSnRK2.7 and HvSnRK2.9 described by Chen *et al.* (2021).

The alignment of whole protein sequences from Arabidopsis and barley was prepared using the MUSCLE algorithm in MEGA 12 (Kumar *et al*., 2024) (Supplementary Fig. S1). The same software was used to build the maximum-Likelihood phylogenetic tree (JTT) for the SnRK proteins with 1000 replicates for bootstrap values (Supplementary Fig. S2). Amino acid alignment visualization and coloring (Clustal) was made using Jalview (Waterhouse *et al*., 2009). The known domains and phosphorylation sites in the SnRK2 proteins were identified according to Belin *et al*. (2006), Yoshida *et al*. (2006) and Soon *et al*. (2012). To generate Arabidopsis lines complementing the lack of *AtOST1* with *HvSnRK2.7* or *HvSnRK2.9* in the *ost1-3* mutant, the vectors for plant transformation were assembled using the GreenGate cloning system (Lampropoulos *et al.*, 2013) and primers shown in Supplementary Table S1. Total RNA from fully developed leaves of Arabidopsis (Col-0) and barley (‘Golden Promise’) plants grown under the same conditions as used for plants in gas exchange experiments [24/19 °C temperature and PAR 250/0 (Arabidopsis) or 750/0 (barley) µmol m^−2^ s^−1^ during day and night, respectively, and 70% relative air humidity, with 12 h /12 h diurnal period] was isolated employing the Spectrum Plant Total RNA kit (Merck KGaA, Darmstadt, Germany). cDNA was prepared with the RevertAid Premium Reverse Transcriptase (Thermo Scientific, Waltham, MA, USA ). The open reading frames (ORFs) of Arabidopsis *OST1* and barley *SnRK2.7* and *SnRK2.9* were amplified from cDNA and cloned into the pGGC vector described in Lampropoulos *et al.*, 2013. Genomic DNA was used for amplification and cloning of the *AtOST1* promoter region into the pGGA vector. Using the destination vector pGGZ004 (Lupanga *et al.*, 2020) as the backbone, the final plasmids for expression of *AtOST1*, *HvSnRK2.7*, and *HvSnRK2.9* under the native *AtOST1* promoter were assembled. These plasmids were introduced into *Agrobacterium tumefaciens* GV3101 for genetic transformation of the *ost1-3* mutant. Using hygromycin B resistance as the selection marker, transgenic lines with single inserts were selected and advanced to homozygosity. The expression of *AtOST1*, *HvSnRK2.7* and *HvSnRK2.9* in the complementation lines was verified using quantitative real-time polymerase chain reaction, qRT–PCR. RNA was extracted as described above using E.Z.N.A Plant RNA Kit (Omega BIO-TEK, Norcross, GA, USA) and treated with DNAse I. cDNA was synthesized using RevertAid H Minus Transcriptase (Thermo Scientific, Waltham, MA, USA). qRT–PCR was performed with the primers indicated in Supplementary Table S1. Expression of the *AtOST1*, *HvSnRK2.7* and *HvSnRK2.9* transcripts was calculated using the qbase+3.2 software (Biogazelle) using the *SAND* (*AT2G28390)* and *YLS8* (AT5G08290) as references for normalization (Han *et al*., 2013).

### Gas exchange experiments with Arabidopsis complementation lines

For Arabidopsis gas exchange experiments, the complementation lines were planted into special pots containing 2:1 (v/v) peat: vermiculite mixture and grown together with Col-0 and *ost1-3* plants in a Percival growth chamber (41AR2cLED; Percival Scientific, Perry, IA, USA) at 24/19 °C during day and night, respectively, and 70% RH, with 12 h /12 h diurnal period. Daytime photosynthetically active radiation (PAR) was ∼250 µmol m^−2^ s^−1^.

During gas exchange experiments, plants were 23–28 days old. Whole-rosette stomatal conductance was recorded with an 8-chamber custom-built temperature-controlled gas-exchange device Jyrkki (PlantInvent Ltd, Tartu, Estonia), which is described in Kollist *et al*. (2007). In VPD experiments, four plants were inserted into the measurement cuvettes and allowed to stabilize at standard conditions (ambient CO_2_ (∼430 ppm), air temperature 24 ±0.5 °C, PAR 250 µmol m^−2^ s^−1^) for at least 60 minutes. Gas exchange of each plant was sampled at 4 min intervals. After stabilization, air VPD was increased from 1.08 ±0.02 kPa to 1.90 ±0.03 kPa and the measurements continued for 40 min. In ABA experiments, eight plants were inserted into the measurement cuvettes for stabilization (ambient CO_2_ of ∼430 ppm, air temperature 24 ±0.5 °C, PAR 260 µmol m^−2^ s^−1^, VPD 0.94 ±0.02 kPa), followed by foliar ABA spraying. During sprayings, plants were removed from the gas exchange cuvettes, sprayed with 5 µM ABA [with 0.012% Silwet L-77 (KCC Corporation, Seoul, South Korea) and 0.05% ethanol] and put back into the cuvettes for the next 47 min of stomatal conductance recordings after ABA treatment. Photographs of plants were taken after the experiments, and their rosette area was calculated using ImageJ 1.54d (Schneider *et al*., 2012). Stomatal conductance for water vapor was calculated with a custom written program as described in Kollist *et al*. (2007).

### Generation of CRISPR barley lines with genetic knock outs of *OST1* homologs

Preparation of the vector for CRISPR/Cas9-based genome editing and Agrobacterium-mediated transformation of barley were conducted using the commercial service at the John Innes Centre (UK). The first exons of barley *SnRK2.7* and *SnRK2.9* were analysed to identify potential sites for genomic editing. The sequences of the selected guides for gRNAs are shown in Supplementary Table S1. The vector for barley transformation contained four cassettes for the expression of gRNAs targeting *HvSnRK2.7* and three cassettes for the expression of gRNAs targeting *HvSnRK2.9* under the TaU3 and TaU6 promoters, as well as the optimized Cas9 cassette (Lawrenson *et al.*, 2024). This vector was used for Agrobacterium-mediated transformation of barley genotype ‘Golden Promise’. In brief, immature embryos were harvested from donor plants, followed by their inoculation with the Agrobacterium strain containing the plasmid for simultaneous editing of *HvSnRK2.7* and *HvSnRK2.9*. Transgenic calli and plants were selected on hygromycin-containing media at the John Innes Centre as described by Hinchliffe and Harwood (2019). Genomic regions of *HvSnRK2.7* and *HvSnRK2.9* in the transgenic T_1_ lines were sequenced to identify the lines with CRISPR-induced mutations. Subsequently, homozygous plants with the confirmed mutations in the target genes and without T-DNA inserts, were identified using PCR with the primers indicated in Supplementary Table S1 and confirmed by sequencing. Two double *snrk2.7 snrk2.9* mutants (lines 15 and 20, hereafter L15 and L20) were studied here. In these lines, a 55 bp deletion was detected at nucleotide position 32 from the coding region start in the first exon of the *SnRK2.7* gene, whereas the *SnRK2.9* sequence was found to have a 1 bp insertion at nucleotide position 20 and a 50 bp deletion at nucleotide position 97 (Supplementary Figs. S3, S4).

### Gas exchange experiments with barley *snrk2.7 snrk2.9* double mutants

For barley gas exchange experiments, double *snrk2.7 snrk2.9* mutants (L15 and L20) and wild-type ‘Golden Promise’ were sown into 1 litre pots (first two, then thinned to one plant per pot) containing peat: vermiculite mixture and grown in a Percival growth chamber (41AR2cLED; Percival Scientific, Perry, IA, USA) at 24/19 °C during day and night, respectively, and 70% RH with 12 h/12 h diurnal period. Two light levels were used to study whether there was any light×genotype interaction on studied gas exchange traits. Daytime photosynthetically active radiation (PAR) was ∼260 and 750 µmol m^−2^ s^−1^ at plant level in low light (LL) and high light (HL) shelves, respectively.

During gas exchange experiments, plants were 14–20 days old. Stomatal conductance (g_s_) and net CO_2_ assimilation rate (A_net_) were recorded with a thermostatically controlled 4-chamber custom-built gas exchange device (Hõrak *et al*., 2017). As a routine, the VPD experiment was performed first, and then after 48 h, the same plants were used in the ABA spraying experiment. All experiments were conducted between 2–7 h after the light period started. In VPD experiments, plants were placed inside gas exchange measurement cuvettes and stomatal conductance, g_s_, was measured at VPD of 0.97±0.02 kPa and 25 °C air temperature, PAR was either 260 or 750 µmol m^−2^ s^−1^ for LL or HL plants, respectively. Gas exchange of each plant was sampled at 8 min intervals. When the stomatal conductance had stabilized (i.e. after 60–90 min), VPD was rapidly increased to 2.08±0.03 kPa for 60 min. After this, low VPD conditions (0.96±0.02 kPa) were restored for the last 60 min, followed by leaf area measurements and returning plants back into the Percival growth chamber. In the ABA experiment, initial conditions for stabilization period were as in the VPD experiment, with VPD values of 1.03±0.02 kPa. For ABA (or mock) spraying, cuvette covers were removed, 20 μM ABA with 0.012% Silwet L-77 (Duchefa) and 0.20% ethanol was sprayed on leaves, cuvette covers were replaced and measurement of stomatal conductance continued for the next 47 minutes. Mock treatment, whose spraying solution contained 0.012% Silwet and 0.20% ethanol, had no effect on stomatal conductance. In wild-type HL plants, pre-treatment and mock-treated g_s_ values were 210.0 (±21.3) and 233.4 (±27.0) mmol m^-2^ s^-1^ (*P*=0.36, GLM Repeated measures ANOVA, *n*=4 plants), respectively, and in L15 HL plants, 379.6 (±39.8) and 352.4 (±35.6) mmol m^-2^ s^-1^ (*P*=0.25; *n*=4 plants), respectively.

Additional gas exchange experiments were performed with HL plants to determine their diurnal courses of g_s_ and A_net_. For this, plants were placed into measurement cuvettes 1 h before the beginning of the dark period and kept there until the next dark period for a full 24 h cycle. The diurnal period during measurements matched that of growth conditions (12 h/12 h).

At the end of each experiment, the leaves enclosed in the measurement cuvettes were photographed for determining leaf area using ImageJ 1.54 d (Schneider *et al*., 2012). When gas exchange experiments were completed, the second leaf of the main shoot was cut for stomatal development (stomatal density, SD, and guard cell length, GCL, of abaxial and adaxial leaf surfaces) measurements and DNA extraction. For SD, the central area of the leaf was sampled and impressions of both the adaxial and abaxial leaf surfaces were made using dental silicone (Speedex light body; Coltene/Whaledent AG, Alstätte, Switzerland) and oranwash L (Zhermack, www.zhermack.com). Secondary imprints made with nail varnish were collected from the silicone and then transferred onto microscope slides with the help of transparent tape. An area of 0.29 mm^2^ from each imprint was photographed under a microscope (Kern OBF 133; KERN & SOHN GmbH; Balingen-Frommern, Germany) at 200× magnification. SD (stomata per mm^2^), and GCL were determined from these images using ImageJ software (Schneider *et al*., 2012).

To determine stomatal aperture widths, leaf impressions (adaxial and abaxial surfaces of the second leaf of the main shoot) were taken and analysed under the microscope as described before for SD determination (five apertures measured and averaged per leaf, *n*=3–6 plants). Here, attached leaves of HL plants growing in the Percival growth cabinet under the conditions specified above were used for imprinting.

After the laboratory experiments, the plants were planted into 3 litre pots and transferred to a greenhouse for propagation and yield determination. Inside the greenhouse, daytime temperature was ∼26 °C and PAR ∼200 µmol m^−2^ s^−1^, whereas relative air humidity ranged between 55% and 70%. The photoperiod of artificial light was 16 h light: 8 h darkness. Plants were kept well-watered, common agricultural practice was used when growing plants in the greenhouse (fertilizer: Osmocote granules; insecticide: Evure; fungicide: Prosaro, according to the manufacturer’s instructions). When physiological maturity was reached, watering was stopped for 14 d, followed by harvesting all ears pot by pot. Grains of mature ears were threshed manually; green ears were discarded. Grain yield per plant was measured after placing the grains in an oven at 32 °C for 72 h.

### Statistical analysis

Analysis of variance (GLM procedure) was used to evaluate the main effects of genotype (G), leaf side and light level (L) on gas exchange traits as well as on grain yield of studied lines, followed by unequal n Tukey post-hoc test (Statistica, version 14.0, StatSoft Inc., Tulsa, OK, USA). The effect of different light levels experienced in the juvenile growth stage was not significant for yield data, and thus, high and low light levels were pooled. Repeated Measures ANOVA with unequal n Tukey HSD as a post-hoc test was used to determine the effects of high VPD and ABA treatments. All effects were considered significant at *P*<0.05.

## Results

### Stomatal regulation of Arabidopsis complementation lines

**Fig. 1.**
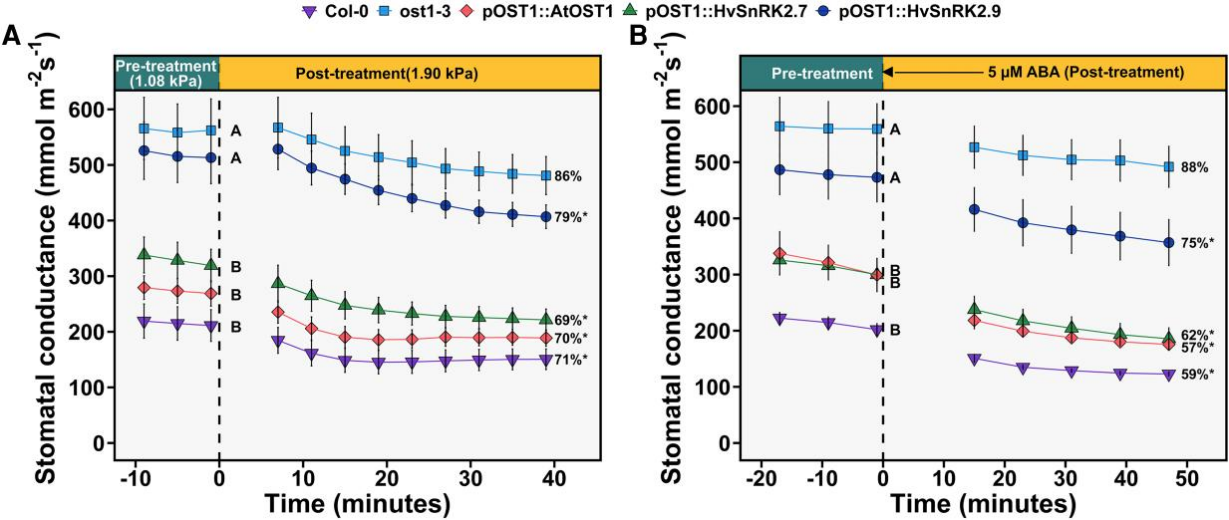
Stomatal responses to high VPD and ABA spraying in Arabidopsis complementation lines. In the complemented lines, barley *HvSnRK2.7* or *HvSnRK2.9* genes, or Arabidopsis *AtOST1* were introduced into the Arabidopsis *ost1-3* background under the control of *OST1* promoter (the lines pOST1::HvSnRK2.7, pOST1::HvSnRK2.9, and pOST1::AtOST1, respectively). The complemented lines were compared with the wild-type Col-0 plants and the *ost1-3* mutant. (A) In high VPD treatment, VPD was changed from 1.08±0.02 kPa to 1.90±0.03 kPa at time 0, g_s_ values represent mean ±SE, *n*=5 plants. Different upper case letters next to the last g_s_ values at low VPD represent statistically significant differences in stomatal conductance between lines (one-way ANOVA and Tukey HSD). Values of g_s_ at the last two time points in pre-treatment and post-treatment were averaged together to get steady-state values of g_s_ at low and high VPDs for statistical comparisons. Percentages on the right show ratios of steady-state g_s_ at high VPD to that at low VPD. The asterisk (*) next to percentage shows that the closure response was statistically significant (Repeated measures ANOVA and Tukey HSD, *n*=5). (B) In the ABA experiment, plants were sprayed with ABA (5 µM) at time 0 (*n*=4–5 plants). All other details are as described for Fig. 1A.

Analysis of the barley genome revealed several proteins, which can be phylogenetically related to Arabidopsis OST1. In our study, we decided to focus on two proteins with the highest similarity to AtOST1, which were identified as SnRK2.7 and SnRK2.9 in Chen *et al.* (2021). The complementation lines were generated where either barley *SnRK2.7* or *SnRK2.9* ORF was introduced into the Arabidopsis *ost1-3* mutant to study whether these proteins can functionally complement OST1 *in planta*. The obtained transgenic lines expressed the *HvSnRK2.7* and *HvSnRK2.9* transcripts at the levels exceeding the expression of *OST1* in wild-type plants (Supplementary Fig. S5). In the complementation lines, barley SnRK2.7 and SnRK2.9 displayed different efficiencies in restoring the OST1 function. Compared with AtOST1, HvSnRK2.7 was as effective in restoring wild-type phenotype: it recovered wild-type level of stomatal conductance and VPD- and ABA-induced closures (Fig. 1A, B; Supplementary Dataset S1). HvSnRK2.9 was less efficient and only partially restored stomatal VPD- and ABA-sensitivities of the respective complementation line (Fig. 1A, B). These results show that SnRK2.7 and SnRK2.9 of barley are functional homologs of Arabidopsis OST1.

### Stomatal regulation and net assimilation rate of barley *snrk2.7 snrk2.9* double mutants

**Fig. 2.**
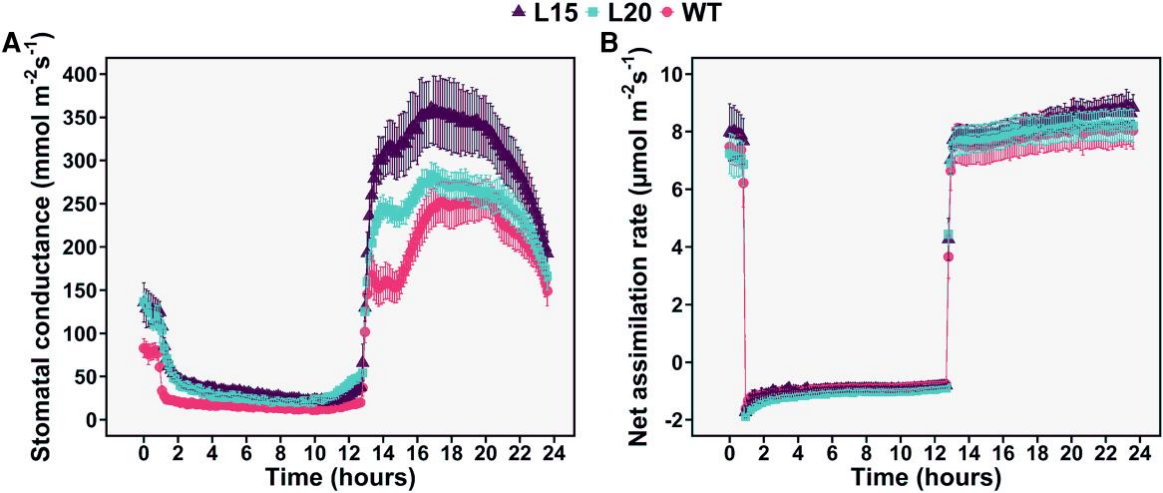
Diurnal courses of stomatal conductance and net assimilation rate in the studied barley lines. Stomatal conductance (A) and net assimilation rate (B) of barley were measured during 24 h (values shown are mean ±SE, *n*=5–6 plants). The light was switched off at 1 h and back on at 13 h. Plants were grown and measured at high light of 750 µmol m^−2^ s^−1^.

Since both HvSnRK2.7 and HvSnRK2.9 complemented Arabidopsis *ost1-3* mutant, although with different efficiencies, we generated and studied a barley double mutant. CRISPR-induced double mutants (individual lines L15 and L20) with mutations in the first exons of the corresponding genes in ‘Golden Promise’, a spring barley cultivar, were generated (Supplementary Fig. S3). In diurnal experiments with HL-grown plants, the g_s_ of all plants increased twice during the day: first, when light was switched on and next, after ∼2 h (Fig. 2A; Supplementary Dataset S1). In the double *snrk2.7 snrk2.9* mutants, the first increase was more pronounced, whereas in wild-type, it was the second increase. Thus, the highest differences in g_s_ values between double mutants and wild-type were detected during the first hours of light period. Since leaf ABA concentrations do not reach minimum values at the onset of light, but a few hours later (Tallman, 2004; Novakova *et al*., 2005), different ABA-sensitivities of studied lines may explain why the second increase of g_s_, likely coinciding with minimum leaf ABA concentration, was larger for ABA-sensitive wild-type plants. When the light was switched off at the end of the day, g_s_ dropped faster in wild-type: after 30 min in darkness, g_s_ decreased to 29%, 52% and 58% of previous values in wild-type, L15 and L20, respectively. Before the lights were switched on in the morning, a pre-dawn stomatal opening was detected only in double mutants (Fig. 2A); this difference could also be explained by their different ABA-sensitivities (Tallman, 2004).

Net CO_2_ assimilation started when light was switched on; A_net_ followed a slight upward trend during the day in all genotypes, and the diurnal and genotypic variation in A_net_ was relatively modest (Fig. 2B).

**Fig. 3.**
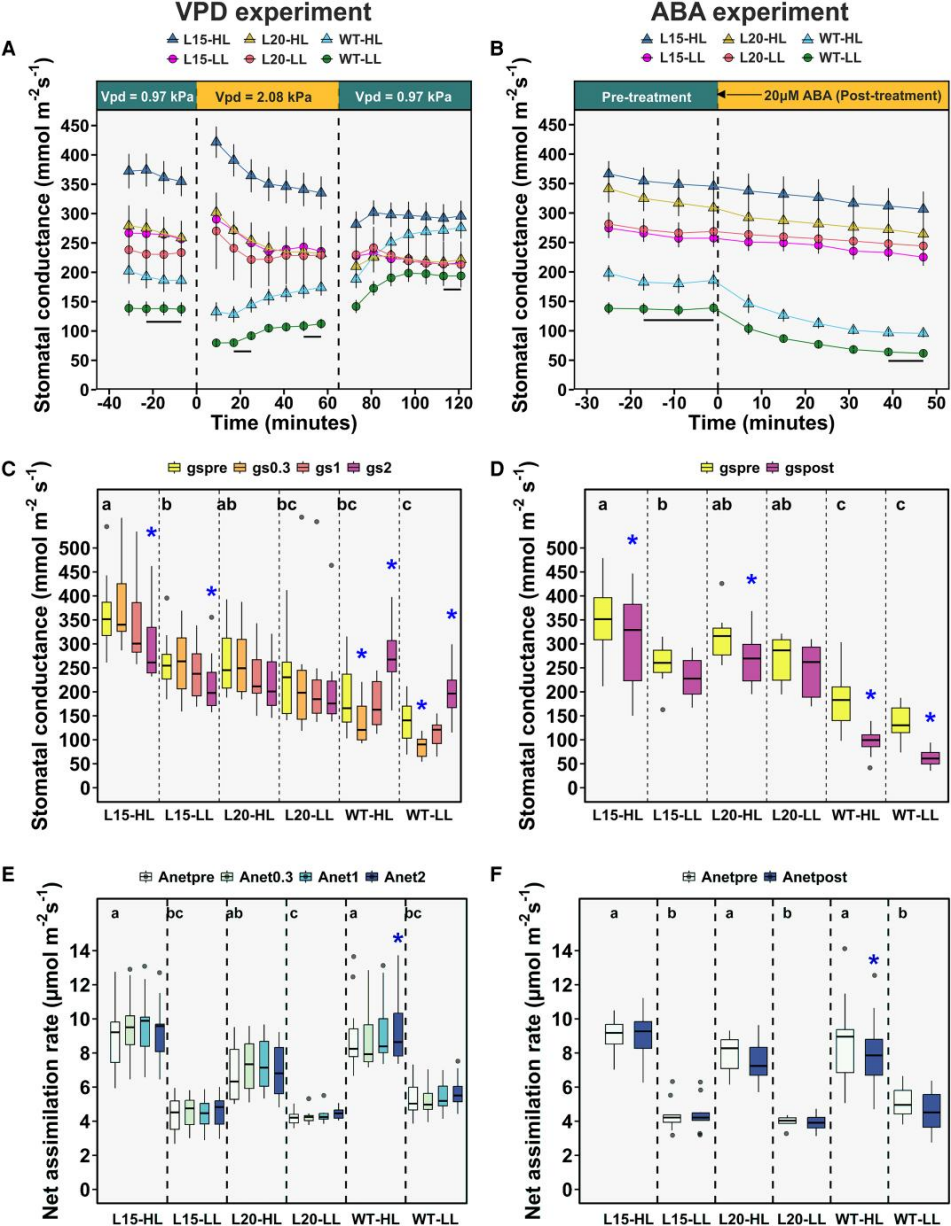
Values of stomatal conductance and net assimilation rate of studied barley lines in VPD and ABA experiments. (A) Dynamic stomatal VPD-responses; VPD was changed from 0.97 to 2.08 kPa at time 0 and then back to 0.96 kPa at 65 min (values shown are mean ±SE, *n*=7–14 plants). Plants were grown and measured under high (HL, 750 µmol m^−2^ s^−1^) or low light (LL, 260 µmol m^−2^ s^−1^) conditions. Lines show which values were averaged to get the values of g_s_pre, g_s_0.3, g_s_1 and g_s_2 shown in Fig. 3C. (B) Dynamic stomatal ABA-responses (values shown are mean ±SE, *n*=7–15 plants). ABA treatment (20 μM) was performed at time 0. Light levels and interpretation of lines as in 3(A). (C) Average values of pre-treatment stomatal conductance (g_s_pre), stomatal conductance 21 and 60 min after VPD increase (g_s_0.3 and g_s_1), and stomatal conductance 60 min after low VPD was restored (g_s_2). (D) Values of pre-treatment stomatal conductance (g_s_pre) and stomatal conductance 47 min after spraying with ABA (g_s_1). (E) Pre-treatment values of A_net_ and A_net_ 21 and 60 min after VPD increase (A_net_0.3 and A_net_1) and 60 min after low VPD was restored (A_net_2). (F) Values of pre-treatment net assimilation rate (A_net_pre) and net assimilation rate 47 min after spraying with ABA (A_net_post) are presented. Different letters above g_s_pre and A_net_pre boxes in panels (C)–(F) indicate statistically significant differences between the genotypes and light levels (*P*<0.05; Table 1); GLM and unequal Tukey HSD), whereas asterisk (*) shows statistically significant difference from g_s_pre or A_net_pre of that line (repeated measures ANOVA and Tukey HSD). Box plots in panels (C)–(F) represent the distribution of values, with the boxes spanning the interquartile range (IQR), and the median indicated by a horizontal line. The whiskers extend to the non-outlier range, and outliers are shown as solid dots.

Next, barley lines were measured for their stomatal responses to high VPD and ABA application. Pre-treatment stomatal conductance (g_s_pre) of double mutants was higher than in wild-type, whereas the main effect of light level on g_s_ was also significant (ANOVA GLM, *P*=0.00062 in VPD experiment and *P*=0.000061 in ABA-experiment; Table 1, Fig. 3A–D). Pre-treatment net assimilation rate, A_net_pre, was significantly affected by genotype and light level as main factors in the VPD experiment, but only by light level in the ABA experiment. Post-hoc tests revealed no significant differences between A_net_pre in genotypes under similar light levels (Table 1, Fig. 3E, F).

**Table 1.** Results of ANOVA GLM for the effects of genotype (G), leaf side and light level (L) on studied stomatal traits and grain yield of barley double mutants and wild-type

Trait	Genotype	Light	Leaf side
g_s_pre, VPD-exp	0.00	0.00	NA
A_net_pre, VPD-exp	0.01	0.00	NA
g_s_pre, ABA-exp	0.00	0.00	NA
A_net_pre, ABA-exp	0.15	0.00	NA
Stomatal density	0.02	0.049	0.00
Guard cell length	0.40	0.34	0.00
Stomatal aperture width	0.00	NA	0.10
Grain yield per plant	0.33	0.56	NA

NA, not available. Genotype×light interaction was never significant.

The stomatal VPD responses of barley *snrk2.7 snrk2.9* double mutants were clearly impaired: upon VPD rise, initial wrong-way response (WWR, as per Buckley, 2019) was detected in all lines, but this passed very quickly in the wild-type due to fast stomatal closure (Fig. 3A). In the double mutants, stomatal response to high VPD was very similar to that of Arabidopsis *ost1-3* (Merilo *et al*., 2018): a slow recovery of stomatal conductance from WWR was detected in L15 and L20, but comparing the respective g_s_pre and g_s_1 values (stomatal conductances before and 60 min after change to high VPD), no statistically significant differences (Repeated measures ANOVA; *P*>0.05) were detected for double mutants (Fig. 3A, C). In wild-type, stomata initially closed but then started to re-open slowly already under high VPD. Similar oscillation-like stomatal behavior has been detected in other plants after switching to high VPD, and can be interpreted as a search for a new equilibrium under changed conditions (Merilo *et al*., 2018; Hsu *et al*., 2021; Zait *et al*., 2024). Stomata of wild-type plants continued with a rapid opening, when VPD was restored to the initial level, whereas this response was not observed in the double mutants (Fig. 3A, C). At the end of the experiment, g_s_ in wild-type was significantly higher (Repeated measures ANOVA, *P*=0.00012 for WT HL and *P*=0.00061 for WT LL) than its g_s_pre (Fig. 3C). In L15 and L20, no increase in g_s_ was measured, when VPD was lowered (Fig. 3A, C); this response to restoration of low VPD is again very similar to Arabidopsis *ost1-3* (Merilo *et al*., 2018).

In response to ABA spraying, statistically significant stomatal closure was detected in wild-type barley plants under high and low light (Repeated measures ANOVA, *P*=0.0001 for WT HL and WT LL), and in L15 and L20 plants under high light (*P*=0.012 and *P*=0.018, respectively), whereas no statistically significant ABA-induced closure was detected in L15 and L20 under low light (*P*=0.18 and *P*=0.76, respectively) (Fig. 3B, D). The ratios of stomatal conductance after ABA treatment to the pre-treatment conductance ranged from 46% to 52% in wild-type LL and HL plants, from 85% to 91% in L20 HL and LL plants, respectively, and was 88% in both L15 LL and HL plants, showing that ABA-responsiveness was impaired in the double mutants.

### Stomatal density, guard cell length and stomatal aperture width of barley *snrk2.7 snrk2.9* double mutants

**Fig. 4.**
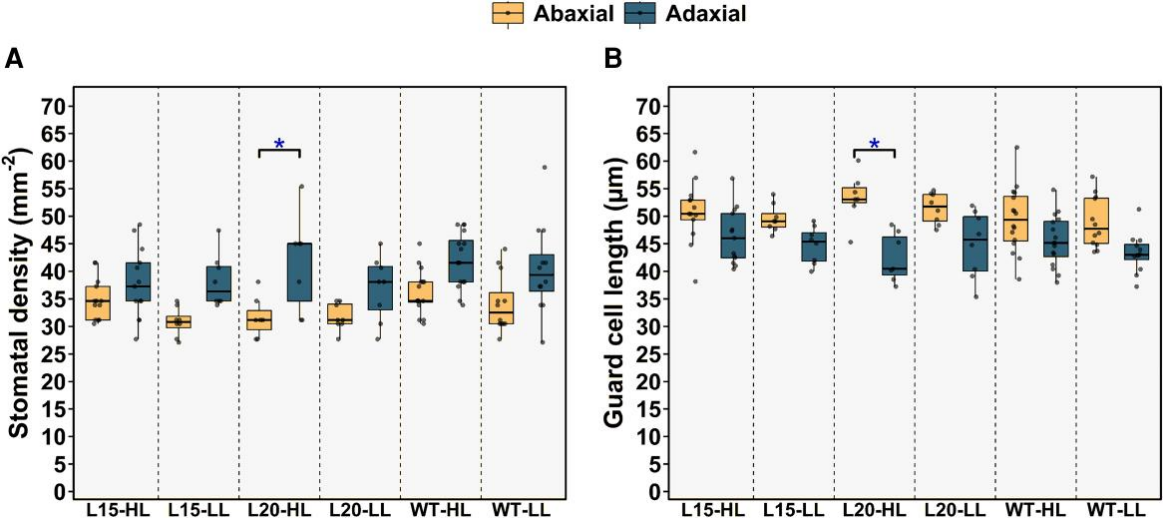
Stomatal density and guard cell length of studied barley lines. (A) Adaxial and abaxial stomatal densities (*n*=7–16 plants). (B) Abaxial and adaxial guard cell lengths (*n*=7–16 plants). The asterisks (*) denote statistically significant differences (GLM with unequal Tukey post hoc test) between adaxial and abaxial leaf surfaces for that line and treatment combination. Box plots in panels (A, B) represent the distribution of values, with the boxes spanning the interquartile range (IQR) and the median indicated by a horizontal line. The whiskers extend to the non-outlier range, and outliers are shown as solid dots.

Next, we studied the differences between wild-type plants and the *snrk2.7 snrk2.9* double mutants in stomatal development. A significant main effect of leaf side (adaxial *versus* abaxial) was detected on SD and GCL: adaxial leaf side always had greater SD with shorter guard cell lengths than abaxial side in all lines and treatment combinations (Fig. 4A, B). SD was significantly affected by genotype and light level as main factors (ANOVA GLM, *P*=0.021 for genotype and *P*=0.049 for light effect), but post-hoc tests revealed no significant differences, when corresponding pairs were compared (Table 1, Fig. 4A). The main effects of line and light level on GCL were non-significant (Table 1, Fig. 4B).

**Fig. 5.**
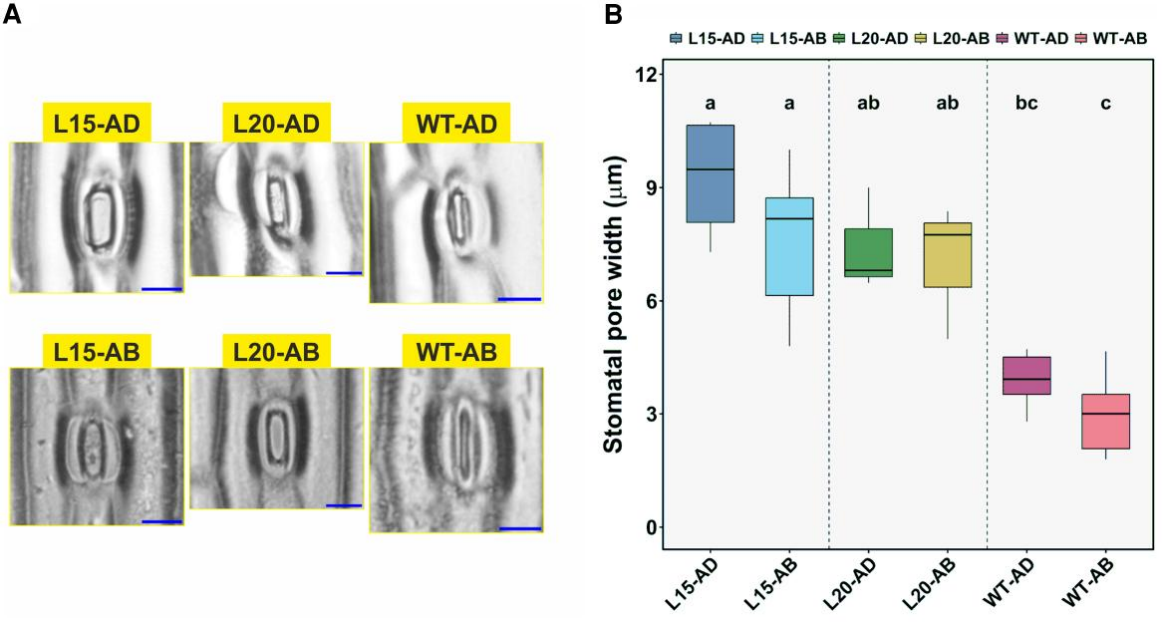
Comparative analysis of stomatal width in high light-grown barley lines. (A) Microscopic images displaying stomatal width variations on AD (adaxial) and AB (abaxial) leaf surfaces across barley double mutants L15, L20, and wild-type (WT) genotypes. The scale bar represents 20 µm. (B) Quantitative measurements of stomatal width (μm); five guard cells were measured per leaf, *n*=3–6 plants per genotype. The data are represented as box plots, where boxes represent the interquartile range with median line, whiskers extend to 1.5×IQR. Different letters indicate significant differences between groups (unequal Tukey’s HSD test, *P*<0.05).

Stomatal imaging of attached leaves showed that in HL-grown double mutants, stomatal aperture width of both leaf sides was significantly larger (ANOVA GLM, *P*=0.000 for genotype effect) compared with wild-type (Fig. 5A, B). Considering the mild (but significant) genotype effect on SD (Fig. 4A), and the strong genotype effect on stomatal aperture width (Fig. 5B), we conclude that differences in g_s_ between genotypes were mostly due to differences in aperture widths.

Total grain yield per plant was 21.1 (±1.2), 22.1 (±1.0) and 23.9 (±1.0) g in wild-type, L15 and L20, respectively, being statistically similar in all genotypes.

## Discussion

The ABA signaling network described for Arabidopsis is conserved in land plants (Hauser *et al*., 2011; Ben-Ari, 2012; Gonzalez-Guzman *et al*., 2014; Kim *et al*., 2014; Kamboj *et al*., 2015; Chen *et al.*, 2017; Collin *et al*., 2020). The module consisting of guard cell anion channel SLOW ANION CHANNEL1 (SLAC1) and its activation by ABA-activated OST1, which is required for active stomatal closure, developed during evolution of early land plants (Lind *et al*., 2015). In this study, we aimed to reveal the role of two barley proteins homologous to Arabidopsis OST1, HvSnRK2.7 and HvSnRK2.9, in stomatal regulation and development, as well as in grain yield formation. Arabidopsis complementation lines, where either HvSnRK2.7 or HvSnRK2.9 was introduced to *ost1-3* background, showed that HvSnRK2.7 statistically completely restored Arabidopsis wild-type phenotype, whereas HvSnRK2.9 improved stomatal sensitivity to VPD and ABA, but had less effect on whole-plant stomatal conductance.

Diurnal measurements of barley stomatal conductance showed that the functional loss of both HvSnRK2.7 and HvSnRK2.9 resulted in higher g_s_ than in wild-type, with differences being highest in the morning hours. Furthermore, the plant genotype was a significant main factor affecting pre-treatment stomatal conductance in VPD and ABA experiments as well (Table 1, Fig. 3). Thus, the SnRK2 loss-of-function barley mutants have higher stomatal conductance as respective mutants in Arabidopsis (Mustilli *et al*., 2002; Merilo *et al*., 2013, 2018). Light level significantly (ANOVA GLM, *P*=0.000 in VPD and ABA-experiments) affected A_net_ in both experiments, but the main effect of genotype on A_net_ was significant only in the VPD experiment (*P*=0.0098; Table 1). Stomatal conductance, but not A_net_, displayed considerable genotypic and daily variation (Figs 2, 3) and a significant increase in g_s_ in double mutants compared with wild-type led to only a minor rise in net assimilation rate (Fig. 3). This indicates that in well-watered conditions, photosynthesis may be limited by non-stomatal factors, and although g_s_ and A_net_ are generally correlated (Wong *et al*., 1979; Hetherington and Woodward, 2003; Jalakas *et al*., 2018b), rise in g_s_ does not necessarily result in boosted photosynthesis and *vice versa* (Hu *et al*., 2006; Hughes *et al*., 2017). Even a modest rise in net assimilation rate, when exerted over the whole canopy (all leaves and ear included) and grain filling period, can have a progressively stimulating effect on grain yield (Parry *et al*., 2011). Grain yields of our lines were, however, statistically similar.

Barley *snrk2.7 snrk2.9* double mutants (L15 and L20) were impaired in VPD- and ABA-induced stomatal regulation, as are Arabidopsis OST1-deficient mutants (Mustilli *et al*., 2002; Merilo *et al*., 2013, 2018; Hsu *et al*., 2021). In Arabidopsis wild-type plants (Col-0 and Ler), g_s_ decreased under high VPD and remained low until low VPD was restored (Merilo *et al*., 2018). In barley wild-type, g_s_ initially significantly dropped under high VPD, but then started to recover, irrespective of high VPD conditions, and continued to increase, when low VPD conditions were restored. It has been shown that leaf hydraulic conductance increases with transpiration rate in well-watered conditions to minimize variation in leaf water potential (Simonin *et al*., 2015). Rise in hydraulic conductance along with high VPD-induced rise in transpiration, together with initial stomatal closure and restoration of leaf water potential, may explain the recovery of g_s_ in wild-type plants.

Stomatal density was significantly influenced by leaf side, genotype and light level, whereas guard cell length was significantly affected only by leaf side (Table 1). Comparing respective pairs by post-hoc test revealed no significant genotype or light level effects. In Arabidopsis, stomatal development was found to be OST1-independent (Jalakas *et al*., 2018a; Yang *et al*., 2022; Tulva *et al*., 2025). Higher light intensity generally results in increased SD in different plants (Miskin and Rasmusson, 1970; Fan *et al*., 2013; Wei *et al*., 2020; Hunt *et al*., 2021); however, in barley, this response depended on experimental light levels (Kubinova, 1991).

Barley simulation models suggest that climate-resilient European barley ideotypes should have higher maximum CO_2_ assimilation rate, radiation use efficiency and water use efficiency (Tao *et al*., 2017), indicating that net assimilation rate needs to increase during future breeding. Drought tolerance and water use efficiency are other key traits to consider, making the breeding of barley for the future a challenging and complex process. In breeding for drought-prone areas, decreased transpiration, which can be achieved by enhanced activation of ABA signaling, e.g. by overexpression of ABA receptors (Santiago *et al*., 2009), is a desirable trait ensuring plant water saving. In greenhouses, hydroponics or paddy fields, where water is not limiting, higher transpiration together with increased net assimilation rate, nutrient uptake and leaf cooling achieved through alleviation of ABA signaling, might be beneficial. For example, pentuple ABA receptor mutant in *Nicotiana benthamiana* showed higher stomatal and mesophyll conductance, net assimilation rate and leaf cooling in well-watered conditions, but as a drawback, was drought-sensitive (Pizzio *et al*., 2024). In paddy field conditions, rice triple ABA receptor mutant showed higher plant biomass and grain yield (Miao *et al*., 2018). However, even though increases in stomatal conductance caused by impaired SLAC1 anion channel resulted in enhanced photosynthesis of rice, *slac1* plants showed signs of leaf water stress under reduced air humidity and decreased growth compared with wild-type in the field (Kusumi *et al*., 2012). Our barley lines with CRISPR/Cas9-edited *SnRK2.7* and *SnRK2.9* displayed higher stomatal conductance and impaired stomatal responsiveness to VPD and ABA. Net CO_2_ assimilation rate of double mutants was much less affected than g_s_ (Table 1, Figs 2, 3C, F), and grain yields of potted well-watered plants were statistically similar in all lines. Further research should address the individual roles of SnRK2.7 or SnRK2.9 in the observed phenotypes of the double mutant. The effect of soil drought and rising atmospheric VPD on the growth of the double mutants versus wild type should be additionally evaluated.

In conclusion, barley double mutants defective in proteins homologous to Arabidopsis OST1 showed higher stomatal conductance and decreased VPD- and ABA-sensitivities compared with wild-type. These results show that as in Arabidopsis, OST1-like kinases control stomatal conductance and sensitivity to ABA and abiotic factors in barley. This increase in g_s_ did not result in boosted photosynthesis, and no statistically significant difference between the lines was detected in grain yield per plant under well-watered conditions.

## Supplementary Material

erag037_Supplementary_Data

## Data Availability

All data supporting the findings of this study are available within the paper and within its supplementary data published online.

## Supplementary data

The following supplementary data are available at *JXB* online.

Fig. S1. The amino acid sequence alignment of A. *thaliana* SnRK2.6 (OST1) and barley SnRK2.7 and SnRK2.9.

Fig. S2. Phylogenetic tree of SnRK2 proteins from barley and *A. thaliana* OST1/SnRK2.6.

Fig. S3. Nucleotide sequence alignments of exon 1 of wild-type barley SnRK2.7 and SnRK2.9 and of double mutants.

Fig. S4. Sequencing of PCR products for the genomic regions containing CRISPR-induced modifications in the L15 and L20 barley lines.

Fig. S5. Calibrated normalized relative quantity levels of *AtOST1*, *HvSnRK2.7* and *HvSnRK2.9* in Col-0, *ost1-3* and complementation lines.

Table S1. The used DNA oligonucleotides in this study.

Supplementary Dataset S1. All data supporting the findings of this study.
