# Supplementary material for: Arabidopsis OST1 homologs of barley are involved in stomatal regulation
Source: J Exp Bot. 2026 Jan 22;77(10):3150–60. doi: 10.1093/jxb/erag037 (PMC13187675; doi:10.1093/jxb/erag037)
Supplement: erag037_Supplementary_Data [file erag037_supplementary_data.zip › Supplemental newer, new.pdf]

Table S1. The used DNA oligonucleotides in this study: PCR primers and guide sequences to target the first exones of *HvSnRK2.7* and *HvSnRK2.9* by CRISPR/Cas9-based genome editing

**Name**	**Sequence**	**Purpose**
pGGC-HvSnRK2.7-Fw	AACAGGTCTCAGGCTCAACAATGGACCGGGCGGCGCTGA	Cloning HvSnRK2.7 into the pGGC vector
pGGC-HvSnRK2.7-Rw	AACAGGTCTCTCTGACATGGCATATACGATCTCCCCGCTGCT	
pGGC-HvSnRK2.9-Fw	AACAGGTCTCAGGCTCAACAATGGCTGGGGCGGCACCGGAT	Cloning HvSnRK2.9 into the pGGC vector
pGGC-HvSnRK2.9-Rw	AACAGGTCTCTCTGACATCGCATACACGATCTCTCCACTGCT	
HvSnRK2.7 noBsaI-Fw	CTTGAACGACGGACTCGACCTTGACG	Removal of the BsaI site from the HvSnRK2.7 sequence
HvSnRK2.7 noBsaI-Rw	CGTCAAGGTCGAGTCCGTCGTTCAAG	
pGGC-AtOST1-Fw	AACAGGTCTCAGGCTCAACAATGGATCGACCAGCAGTGAG	Cloning AtOST1 into the pGGC vector
pGGC-AtOST1-Rw	AACAGGTCTCTCTGACATTGCGTACACAATCTCTC	
pGGA-pAtOST1-Fw	AACAGGTCTCAACCTCTCTAGAAGAGTGTATTGGG	Cloning the pAtOST1 promoter into the pGGA vector
pGGA-pAtOST1-Rw	AACAGGTCTCTTGTTTCTTTCCTCTGTCTCTTTGC	
pAtOST1-noBSAI-Fw	AACAGGTCTCGGGACTCTGAAAACGTCTCT	Removal of the BsaI site from the AtOST1 sequence
pAtOST1-noBSAI-Rw	AACAGGTCTCAGTCCCGTGAAGTATCTAACC	
HvSnRK2.7-PCR-868-Fw	CTGGAGCTCATCATCAGCTAATTAAACT	Amplification of the HvSnRK2.7 targeted by CRISPR
HvSnRK2.7-PCR-1628-Rw	GACCAATTTAGCAGCTGCCGTA	
HvSnRK2.7-seq-891-Fw	CAGCAGGCCAAGGCTTCT	Primers for sequencing of the PCR fragment
HvSnRK2.7-seq1541-Rw	CTGAGAGCAGCTGCTGGAA	
HvSnRK2.9-PCR-1674-Fw	GCCGAAAGATGACGTCCAATAG	Amplification of the HvSnRK2.9 targeted by CRISPR
HvSnRK2.9-PCR-2283-Rw	TACACCGTCCGAATCGAACA	
HvSnRK2.9-seq1848-Fw	TGAGCTCGCCGTCGATTC	Primers for sequencing of the PCR fragment
HvSnRK2.9-seq2200-Rw	AAACAGGGCGGAGTCGATT	
pZmUBQ1-PCR-Fw	TGTGGTTGGGCGGTCGTTCATT	Detecting plants without the pZmUBQ1::Cas9 cassette
pGGA-pZmUBQ1-Rw	AAAAGGTCTCATGTTTCCTGCAGAAGTAACACCAAACAACAGG	
HvSnRK2.7-qPCR-Fw	ACCAGTTCTTGAACGACGGa	qPCR for the HvSnRK2.7 transcript
HvSnRK2.7-qPCR-Rw	TATACGATCTCCCCGCTGCT	
HvSnRK2.9-qPCR2-Fw	CCACCCGCTTGTTCTCGTAT	qPCR for the HvSnRK2.9 transcript
HvSnRK2.9-qPCR2-Rw	ACACGATCTCTCCACTGCTG	
AtOST1-qPCR-Fw	TGCTTACATCGCTCCTGAGG	qPCR for the AtOST1 transcript
AtOST1-qPCR-Rw	CAGAGTAACCCCACAAGACCA	
SAND-Fw	CAGACAAGGCGATGGCGATA	Reference for qPCR
SAND-Rw	GCTTTCTCTCAAGGGTTTCTGGGT	
YLS8-Fw	TTACTGTTTCGGTTGTTCTCCATTT	Reference for qPCR
YLS8-Rw	CACTGAATCATGTTCGAAGCAAGT	
HvSnRK2.7-guide sequence#1	ATTATGGGCATGTCCATCCC	Expressed under the TaU6 promoter
HvSnRK2.7-guide sequence#2	CTCGTCAAGGACATCGGCTC	Expressed under the TaU3 promoter
HvSnRK2.7-guide sequence#3	CTCATGCGCAACCGCGCCGA	Expressed under the TaU6 promoter
HvSnRK2.7-guide sequence#4	AGCGCATCTGCAACGCCGGC	Expressed under the TaU3 promoter
HvSnRK2.9-guide sequence#5	ACCGTCAGCGCCGCCCGATC	Expressed under the TaU3 promoter
HvSnRK2.9-guide sequence#6	TGATCGGCATGTCCATGCCC	Expressed under the TaU6 promoter
HvSnRK2.9-guide sequence#7	TACGAGCTGGTGCGGGACAT	Expressed under the TaU3 promoter

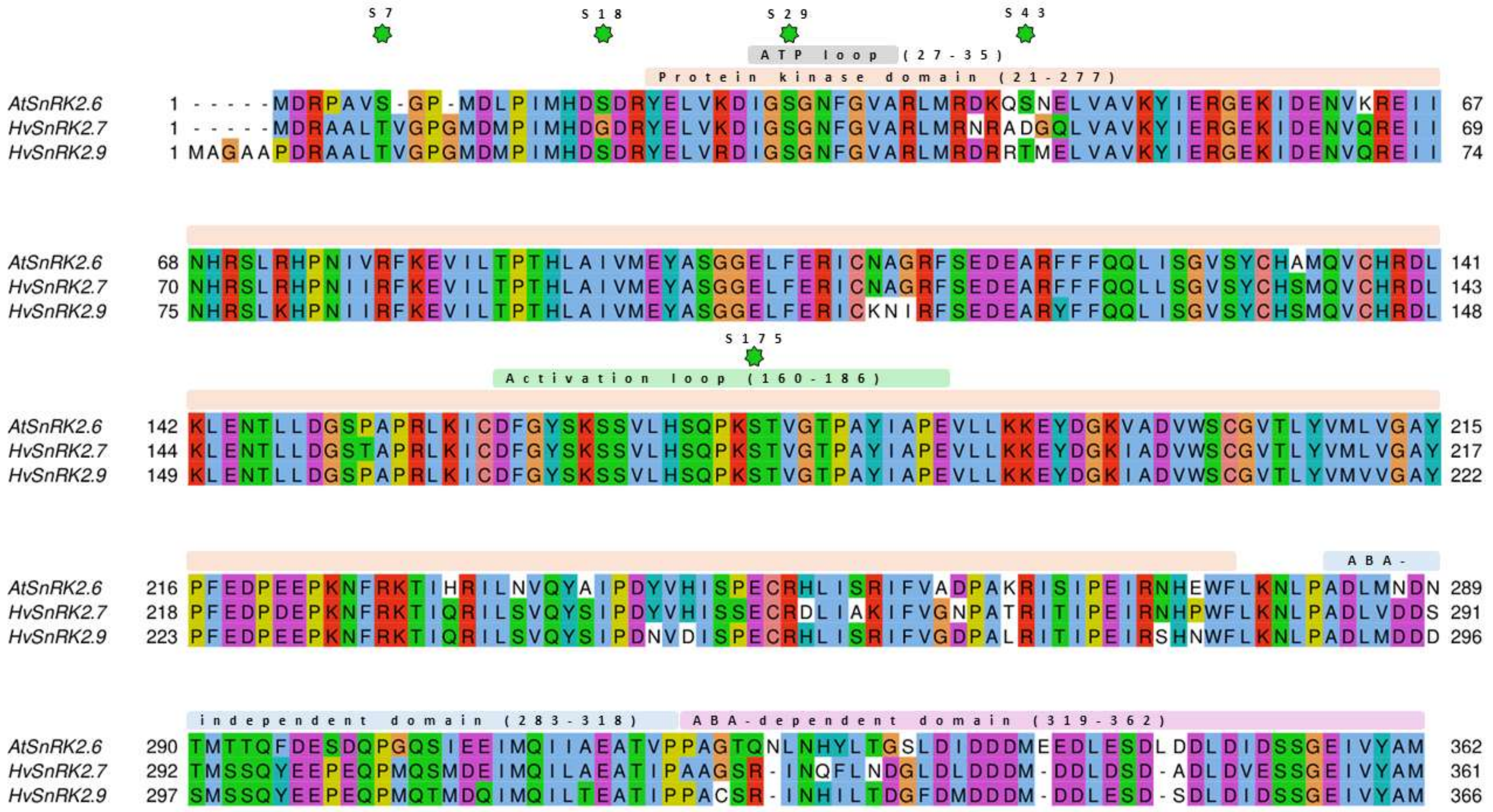

Figure S1. The amino acid sequence alignment of A. *thaliana* SnRK2.6 (OST1) and barley SnRK2.7 and SnRK2.9 (MUSCLE alignment, Clustal coloring). ATP-loop, protein kinase domain, activation loop, ABA-independent and ABA-dependent domains are indicated. The phosphorylation sites known for AtOST1 are indicated by green heptagrams.

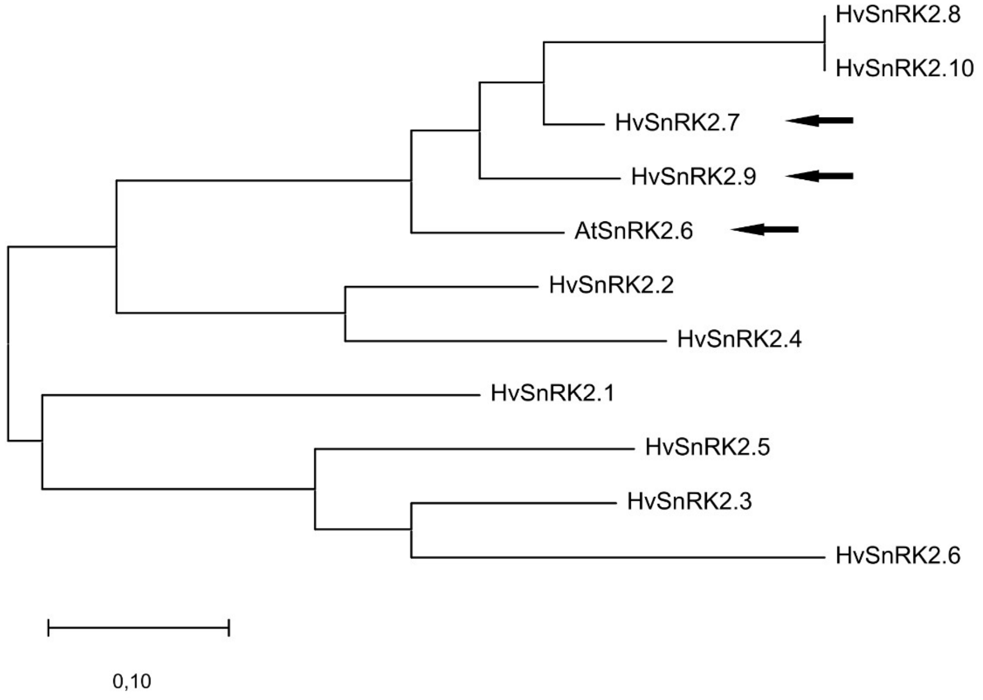

Figure S2. Phylogenetic tree of SnRK2 proteins from barley and *A. thaliana* OST1/SnRK2.6. The tree was built based on the MUSCLE alignment (Figure S1). SnRK2s relevant for this study are indicated by arrows.

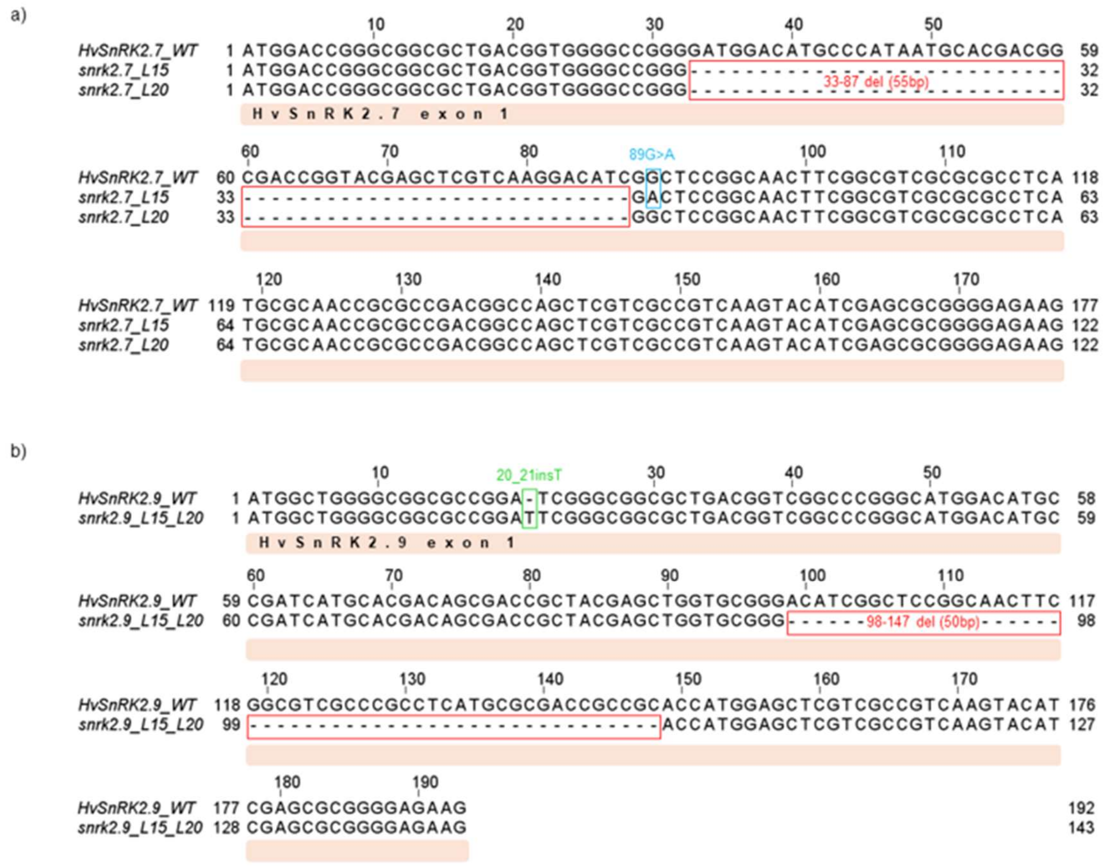

Figure S3. Nucleotide sequence alignments of exon 1 of wild-type barley SnRK2.7 (a) and SnRK2.9 (b) with their respective modified variants in double mutants. Substitutions are marked in blue, insertions in green and deletions in red.

Deletion

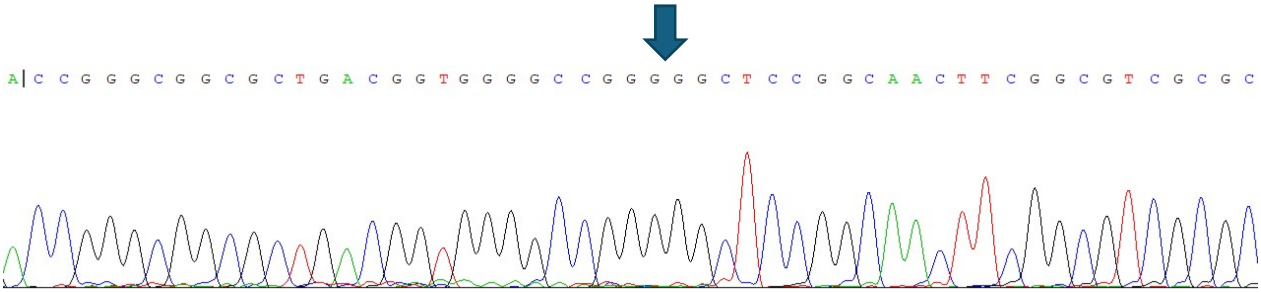

OST1.1 in L20 (the deletion site)

One nucleotide insert

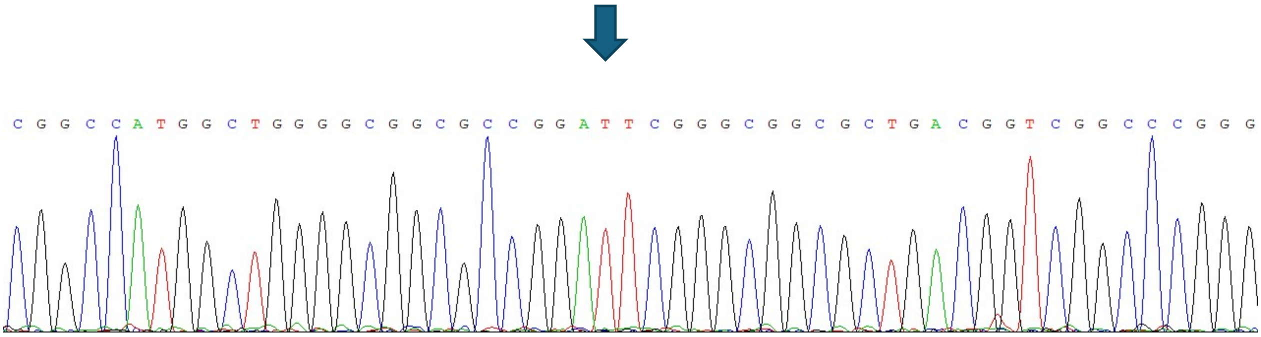

OST1.3 in L20 (one nucleotide insert)

Deletion

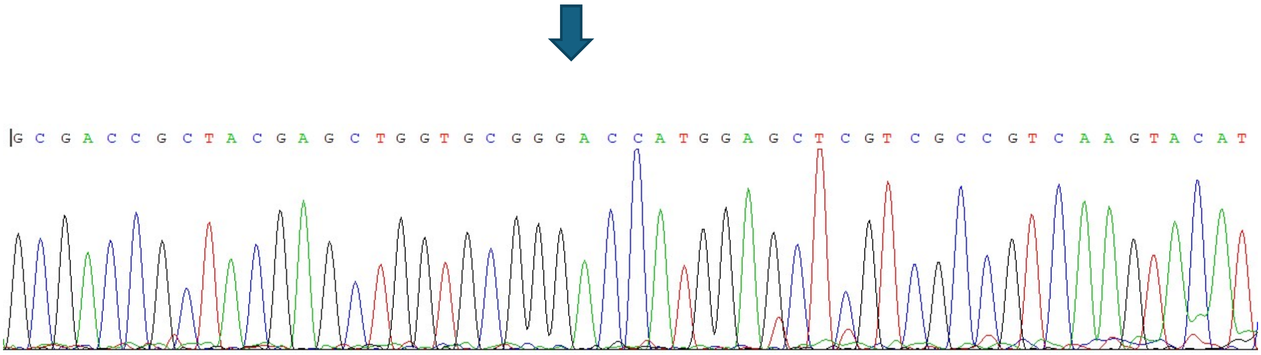

OST1.3 in L20 (the deletion site)

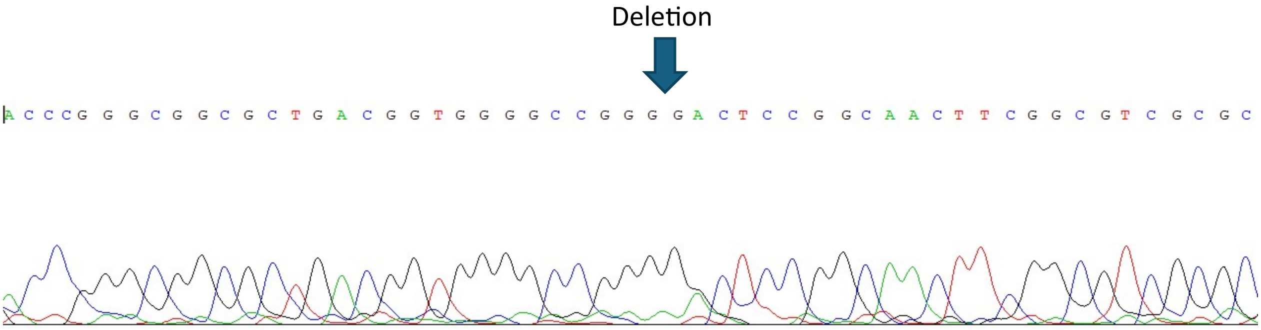

OST1.1 in L15 (the deletion site)

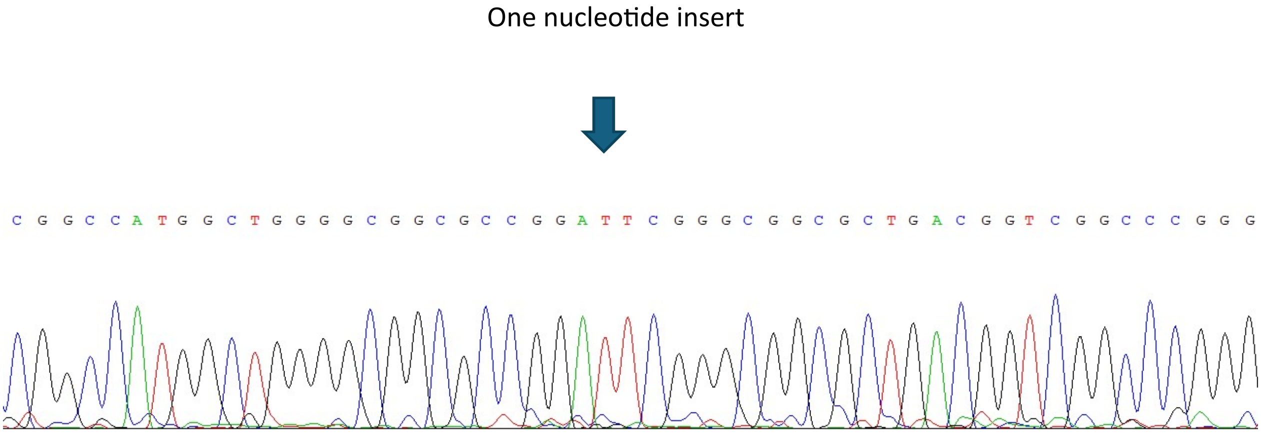

OST1.3 in L15 (one nucleotide insert)

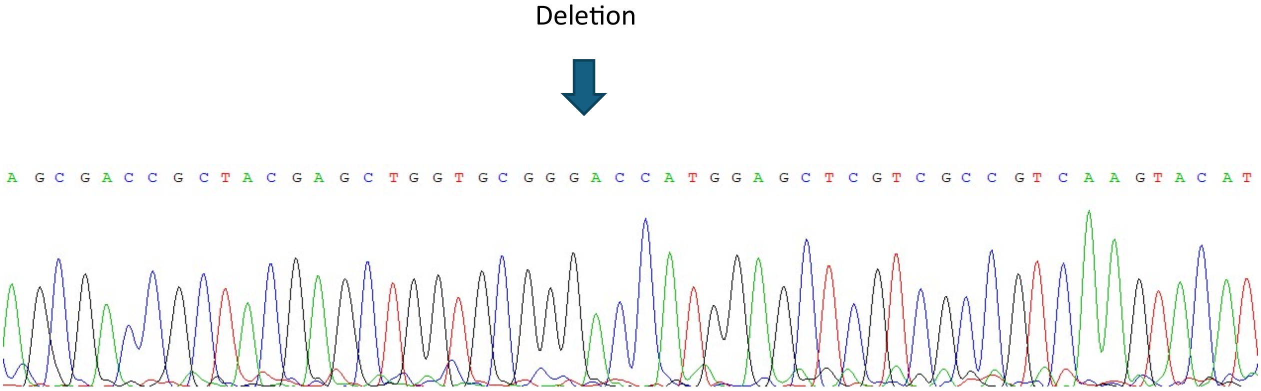

OST1.3 in L15 (the deletion site)

Fig. S4. Sequencing of PCR products for the genomic regions containing CRISPR-induced modifications in the L15 and L20 barley lines. A 55 bp deletion was detected at nucleotide position 32 from the coding region start in the first exon of the *SnRK2.7* gene, whereas the *SnRK2.9* sequence was found to have a 1 bp insertion at nucleotide position 20 and a 50 bp deletion at nucleotide position 97.

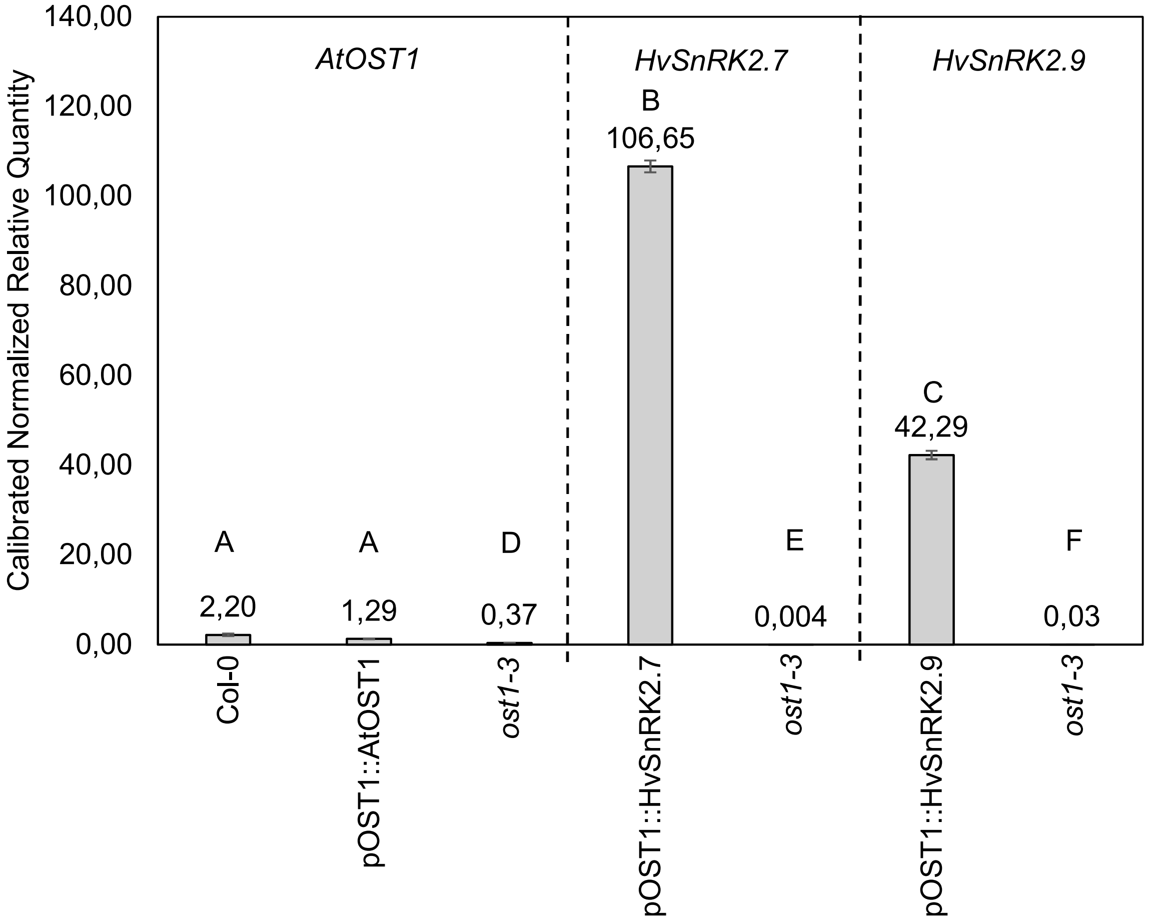

Figure S5. Calibrated normalised relative quantity levels of *AtOST1*, *HvSnRK2.7* and *HvSnRK2.9* in Col-0, *ost1-3* and complementation lines. Statistical groups marked with different letters. Mean ± SEM, n=3-4 technical repeats. *HvSnRK2.7* and *HvSnRK2.9* are highly expressed in the respective Arabidopsis complementation lines, whereas the expression level of *AtOST1* is less. The expression levels of *AtOST1*, *HvSnRK2.7* and *HvSnRK2.9* in Arabidopsis *ost1-3* mutant are negligible.
